# 4,3-α-Glucanotransferase, a novel reaction specificity in glycoside hydrolase family 70 and clan GH-H

**DOI:** 10.1038/srep39761

**Published:** 2017-01-06

**Authors:** Joana Gangoiti, Sander S. van Leeuwen, Gerrit J. Gerwig, Stéphane Duboux, Christina Vafiadi, Tjaard Pijning, Lubbert Dijkhuizen

**Affiliations:** 1 Microbial Physiology, Groningen Biomolecular Sciences and Biotechnology Institute (GBB), University of Groningen, Nijenborgh 7, 9747 AG Groningen, The Netherlands; 2 Nestlé Research Center, Vers-Chez-Les-Blanc, Lausanne, Switzerland; 3 Biophysical Chemistry, Groningen Biomolecular Sciences and Biotechnology Institute (GBB), University of Groningen, Nijenborgh 7, 9747 AG Groningen, The Netherlands

## Abstract

Lactic acid bacteria possess a diversity of glucansucrase (GS) enzymes that belong to glycoside hydrolase family 70 (GH70) and convert sucrose into α-glucan polysaccharides with (α1 → 2)-, (α1 → 3)-, (α1 → 4)- and/or (α1 → 6)-glycosidic bonds. In recent years 3 novel subfamilies of GH70 enzymes, inactive on sucrose but using maltodextrins/starch as substrates, have been established (e.g. GtfB of *Lactobacillus reuteri* 121). Compared to the broad linkage specificity found in GSs, all GH70 starch-acting enzymes characterized so far possess 4,6-α-glucanotransferase activity, cleaving (α1 → 4)-linkages and synthesizing new (α1 → 6)-linkages. In this work a gene encoding a putative GH70 family enzyme was identified in the genome of *Lactobacillus fermentum* NCC 2970, displaying high sequence identity with *L. reuteri* 121 GtfB 4,6-α-glucanotransferase, but also with unique variations in some substrate-binding residues of GSs. Characterization of this *L. fermentum* GtfB and its products revealed that it acts as a 4,3-α-glucanotransferase, converting amylose into a new type of α-glucan with alternating (α1 → 3)/(α 1 → 4)-linkages and with (α1 → 3,4) branching points. The discovery of this novel reaction specificity in GH70 family and clan GH-H expands the range of α-glucans that can be synthesized and allows the identification of key positions governing the linkage specificity within the active site of the GtfB-like GH70 subfamily of enzymes.

Glycoside hydrolase family 70 (http://www.cazy.org/) initially was established to accommodate glucansucrase enzymes converting sucrose into α-glucan polysaccharides, exclusively found in lactic acid bacteria, especially in the genera *Leuconostoc, Streptococcus, Lactobacillus, Weissella* and *Oenococcus*^1^,^2^. Depending on the GS specificity, structurally different α-glucans are formed. Initially mostly glucansucrase enzymes synthesizing α-glucans with (α1 → 3)- (mutan) or (α1 → 6)-linkages (dextran) were characterized^3^,^4^,^5^,^6^; in recent years also various α-glucans with (α1 → 2)- or (α1 → 4)-linkages have been identified as glucansucrase products^7^,^8^,^9^. A few glucansucrase enzymes have been characterized that produce α-glucans with alternating (α1 → 3)/(α1 → 6)-linkages (alternan) (e.g. alternansucrase of *Leuconostoc mesenteroides* NRRL B-1355)^10^ or (α1 → 4)/(α1 → 6)-linkages (reuteran) (e.g. reuteransucrase of *Lactobacillus reuteri* 121)^8^,^9^. Also, branching points may be introduced, either by the same enzyme^11^ or by separate (α1 → 2)- or (α1 → 3)-branching enzymes of *L. mesenteroides* strains^12^,^13^. GSs thus are able to synthesize all four possible linkage types of α-glycosidic bonds [(α1 → 2), (α1 → 3), (α1 → 4) and (α1 → 6)], but certain combinations of glycosidic linkages have been never found within the same α-glucan. For example, no wild-type glucansucrase enzymes synthesizing α-glucans with (α1 → 3) plus (α1 → 4)-linkages have been reported so far. The α-glucans produced by GSs also differ in their degree of branching, molecular mass, and conformation^2^. Such differences result in α-glucans showing different functional properties with diverse and promising industrial applications.

Family GH70 GS enzymes (acting on sucrose) are evolutionary related to family GH13 α-amylase enzymes (acting on maltodextrins/starch), constituting clan GH-H^14^,^15^,^16^,^17^. Due to their evolutionary relatedness, GH70 and GH13 family enzymes display similarities in their sequence and structure, and use an α-retaining double displacement catalytic mechanism^14^,^18^. Both families have a catalytic (β/α)_8_ barrel structure in their proteins, with domains A, B and C, and an active site with 4 conserved regions regarded as sequence fingerprints for the individual enzyme specificities^18^,^19^. However, GSs also exhibit unique features. In family GH13 the order of these 4 conserved regions is I-II-III-IV. In contrast, in GS enzymes this (β/α)_8_ barrel is circularly permuted, which results in the conserved region order II-III-IV-I. Moreover, GSs possess two extra and unique domains IV and V^12^,^20^,^21^,^22^. Besides, GSs present a “U-fold” domain structure in which 4 (domains A, B, IV and V) of the 5 domains are built up from two discontinuous segments of the polypeptide chain^12^,^20^,^21^. During their evolution from GH13, the GH70 enzymes appear to have undergone a sequence of gene rearrangements that resulted in this unusual, circularly permuted domain organization^23^.

In recent years several maltodextrins/starch converting enzymes have been identified within the GH70 family supporting the evolutionary relatedness of GH13 and GH70 families^23^,^24^,^25^,^26^. Firstly, it was found that *L. reuteri* 121 produced a GS-like enzyme that was inactive on sucrose. The gene encoding this enzyme was found upstream of the gene encoding the glucansucrase GtfA and was designated as *gtfB*. Instead of sucrose, the *L. reuteri* 121 GtfB acts on maltodextrins and starch substrates, cleaving (α1 → 4)-linkages from the non-reducing end of the donor substrate, and synthesizing new (α1 → 6)-linkages on the non-reducing end of the product (4,6-α-glucanotransferase activity, 4,6-α-GTase)^24^,^27^,^28^. This results in the synthesis of products with linear chains of (α1 → 6)- and (α1 → 4)-linkages (isomalto/maltopolysaccharides, IMMP)^18^. Later on, 2 more GtfB homologues were characterized displaying the same substrate and product specificity^25^. A total of 46 related GtfB type of enzymes are currently available in databases, constituting a new GH70 subfamily; with 4 exceptions they are all found in the genus *Lactobacillus*. Following the annotation of many new genome sequences, a few family GH70 enzymes also were found in non-LAB genera. Previously we have characterized the GtfC enzyme of *Exiguobacterium sibiricum* 255–15^23^ and the GtfD enzyme of *Azotobacter chroococcum* NCIMB 8003^26^. Both of these enzymes are inactive with sucrose and active with maltodextrins/starch, displaying 4,6-α-GTase activity that resulted in synthesis of isomalto/malto oligosaccharides (IMMO) (GtfC)^23^ and in a reuteran type of α-glucan (GtfD)^26^. Surprisingly, the domain organization in GtfC and GtfD resembles that of GH13 enzymes, with a nonpermuted order of conserved regions I-II-III-IV, and lacking domain V found in other GH70 enzymes. GtfC and GtfD represent 2 additional GH70 subfamilies and structurally appear to be very interesting evolutionary intermediates between GH13 α-amylase and GH70 glucansucrase enzymes, allowing further analysis of the evolutionary origins and differentiation of the (sub) families in clan GH-H (http://www.cazy.org/).

Based on the discovery of GtfB, GtfC and GtfD type of enzymes, it appears that 4,6-α-GTase is a common activity within the GH70 family. However, the lack of 3D structural information and of α1 → 2 and α1 → 3 synthesizing maltodextrins/starch utilizing type of GH70 enzymes has limited our understanding of the structural features determining this different substrate and product specificity. For GSs, the crystal structure of GTF180-ΔN with a bound maltose acceptor combined with site-directed mutagenesis experiments revealed that the linkage specificity is controlled by residues forming the acceptor substrate binding subsites +1/+2^20^,^29^,^30^,^31^,^32^. Despite their different domain organization, GtfB, GtfC, and GtfD 4,6-α-GTase enzymes display high conservation in their motifs I to IV, particularly in residues forming the acceptor substrate binding subsites in GSs^23^,^26^. Interestingly, a large number of these residues conserved in these 4,6-α-GTases differ in the GSs group of enzymes allowing the 4,6-α-GTase activity to be predicted by the analysis of the signature motifs I to IV.

In this work, annotation of the *Lactobacillus fermentum* NCC 2970 genome sequence resulted in identification of a GtfB-like GH70 enzyme displaying unique variations in some of the residues in conserved regions II and IV, contributing to the active site donor/acceptor substrate binding subsites. We therefore decided to biochemically characterize this *L. fermentum* GtfB enzyme, including a detailed characterization of its products. This revealed that it possesses 4,3-α-glucanotransferase (4,3-α-GTase) activity with maltodextrins/starch, a novel reaction specificity in family GH70 and clan GH-H. The *L. fermentum* GtfB thus represents a very interesting enzyme for investigation of structural features determining the linkage specificity in the GtfB-like GH70 subfamily of enzymes.

## Materials And Methods

### *Lactobacillus fermentum* NCC 2970 genome sequence analysis

The *Lactobacillus fermentum* NCC 2970 genome sequence has been obtained previously (GenBank accession no. CP017151) and was analyzed for the presence of carbohydrate active enzymes using the dbCAN database for automated Carbohydrate-active enzyme Annotation^33^. Hits having an E-Value below 1^E-18^ and a coverage above 0.35 were considered. Exploration of the genome and its gene content was performed using the WallGene software^34^.

### *L. fermentum* NCC 2970 GtfB protein sequence analysis

BLAST analysis of the *L. fermentum* NCC 2970 genome using the *L. reuteri* 121 *gtfB* gene (GenBank accession no. AAU08014.2) as query sequence revealed the presence of a single gene encoding a GH70 protein (CDS0277). Sequences showing similarity to the *L. fermentum* NCC 2970 GH70 protein sequence (GenBank accession no. AOR73699) were found using NCBI BLASTp searches (http://www.ncbi.nlm.nih.gov/BLAST/) against the non-redundant protein sequence database. Multiple amino acid sequence alignments were made with Clustal W2 using the Jalview 2 desktop application. The presence of a signal peptide was analyzed using the Signal P4 server. Subcellular localization of the *L. fermentum* GtfB protein was predicted using CELLO v.2.5: subCELlular LOcalization predictor (http://cello.life.nctu.edu.tw/). The theoretical *M_w_* (molecular weight) of the GtfB protein was predicted on ExPASy Compute pI/*M_w_* (http://web.expasy.org/compute_pi/).

Phylogenetic analysis was performed using MEGA version 6^35^ with a total of 72 amino acid sequences corresponding to representative characterized GH70 proteins indexed in CAZy and GtfB-like protein sequences identified via BLASTp. Sequences were aligned by MUSCLE, using default parameters. A phylogenetic tree was constructed by the Maximum Likelihood method based on the JTT matrix model using MEGA6. Partial deletion of the positions containing alignment gaps and missing data was conducted. Statistical confidence of the inferred phylogenetic relationships was assessed by performing 1,000 bootstrap replicates.

### Cloning of the *L. fermentum* NCC 2970 *gtfB* gene

The DNA fragment coding for an N-terminally truncated version of the GtfB protein (amino acids 616–1593) was amplified from *L. fermentum* NCC 2970 chromosomal DNA using Phusion DNA polymerase (Finnzyme, Helsinki, Finland) and cloned into a modified pET15b vector using ligation-independent cloning (LIC). The primers used for amplifying the N-terminally truncated *gtfB* gene derivative incorporated 5′ extensions (underlined) to facilitate the LIC cloning, and were: Forward CAGGGACCCGGTTTTGGTAAAGATGGTCGGATTG and Reverse CGAGGAGAAGCCCGGTTAATTGTCTTCAATATTAGCATAATAATC. The resulting PCR product was purified from the agarose band and digested in the presence of dATP, with the 3′ to 5′ exonuclease activity of the T4 DNA polymerase (New England Biolabs). In parallel, the pET15b/LIC vector was digested with KpnI, isolated from gel and then treated with T4 DNA polymerase (New England Biolabs) in the presence of dTTP. The treated pET15b/LIC vector and the amplicon were mixed together in a 1:4 molar ratio, and the mixture was used to transform *Escherichia coli* DH5α cells (Phabagen). This resulted in a *gtfB-ΔN* construct containing an N-terminal His6-tag cleavable by a 3 C protease. The constructed expression vector pET15b/*gtfB-ΔN* was transformed into host *E. coli* BL21 Star (DE3). The gene sequence was verified by nucleotide sequencing (GATC, Cologne, Germany).

### Enzyme expression and purification

For expression of the *L. fermentum* NCC 2970 GtfB-ΔN enzyme, an *E. coli* star BL21 (DE3) overnight culture, transformed with pET15b/*gtfB-ΔN* was diluted 1:100 into fresh LB broth supplemented with ampicillin (100 μg ml^−1^) and grown at 37 °C and 220 rpm until the optical density at 600 nm reached approximately 0.4. The temperature for culturing was then decreased to 16 °C, and the inducer isopropyl-β-D-1-thiogalactopyranoside was added to a final concentration of 0.1 mM. After 20 h, cells were harvested by centrifugation (10,000 g × 20 min), and subsequently disrupted with B-PER lysis reagent in accordance to the protocol described by the manufacturer (Thermo Scientific, Pierce). The recombinant GtfB protein was isolated from the cell-free extract by His-tag affinity chromatography using Ni^2+^-nitrilotriacetate (Ni-NTA) as column material (Sigma-Aldrich). After washing the column with 25 mM Tris-HCl (pH 8.0), 1 mM CaCl_2_, bound proteins were eluted with 200 mM imidazole in the same buffer and the imidazole was removed by use of a stirred ultrafiltration unit (Amicon, Beverly, MA) with a 30,000 molecular weight cut off. For further purification, the protein was loaded on a 1 ml-HiTrap column (GE Healthcare) and eluted (at a 1 ml/min flow rate) using a linear gradient of NaCl (from 0 to 1 M) in 20 mM Tris-HCl buffer (pH 8.0), containing 1 mM CaCl_2_. Fractions of 1 ml were collected using an Äkta fast protein liquid chromatograph (FPLC; GE Healthcare, Uppsala, Sweden). The buffer was exchanged by ultrafiltration (YM30 membranes; Millipore, Billerica, MA). The purification progress was assessed by SDS-PAGE analysis of the fractions, and the protein concentrations were determined using a Nanodrop 2000 spectrophotometer (Isogen Life Science, De Meern, The Netherlands).

### Enzyme activity assays

The initial total activity of the *L. fermentum* NCC 2970 GtfB-ΔN enzyme was determined by the amylose-iodine method as described before^23^,^27^. The decrease in absorbance of the α-glucan-iodine complex resulting from transglycosylation and/or hydrolytic activity was monitored at 660 nm for 14 min at 40 °C. The reaction mixture contained 0.125% (w v^−1^) amylose V (AVEBE, Foxhol, The Netherlands), 2.5 μg ml^−1^ of enzyme, 25 mM sodium acetate (pH 5.5) and 1 mM CaCl_2_. One unit of activity was defined as the amount of enzyme converting 1 mg of substrate per min.

The effect of pH on enzyme activity was determined at 40 °C by varying the pH between 3.5 and 8.0. Sodium citrate buffer (25 mM) was used for pH values between 3.5 and 7.0, and sodium phosphate buffer (25 mM) for pH values between 7.0 and 8.0. The optimal temperature was determined in 25 mM sodium citrate buffer (pH 5.5), 1 mM CaCl_2_, at temperatures ranging from 30 to 65 °C. The effect of temperature on GtfB-ΔN stability was determined by incubating the enzyme at a concentration of 0.1 mg ml^−1^ in 20 mM Tris-HCl buffer (pH 5.5) with 1 mM CaCl_2_ at temperatures from 30 to 55 °C for 10 min. Samples were then immediately cooled to 4 °C, and the residual activity was measured under the standard reaction conditions in 25 mM sodium citrate (pH 5.5) with 1 mM CaCl_2_ at 40 °C.

### Substrate utilization by *L. fermentum* NCC 2970 GtfB

*L. fermentum* NCC 2970 GtfB-ΔN (25 μg ml^−1^) was separately incubated with 25 mM sucrose (Acros), nigerose (Sigma-Aldrich), panose (Sigma-Aldrich), isomaltose (Sigma-Aldrich), isomaltotriose (Sigma-Aldrich), isomaltopentaose (Carbosynth), malto-oligosaccharides (MOS) with degrees of polymerization (DP) 2–7, and 0.6% (w v^−1^) amylose V (AVEBE, Foxhol, The Netherlands), potato starch (Sigma-Aldrich) and amylopectin (Sigma-Aldrich). All reactions were performed in 25 mM sodium acetate buffer (pH 5.5) with 1 mM CaCl_2_ at 37 °C for 24 h. Reactions were stopped by 6 min of incubation at 100 °C. The progress of the reactions was monitored by thin-layer chromatography (TLC) and/or high-performance-anion-exchange chromatography (HPAEC).

### Thin Layer Chromatography and High Performance Anion Exchange Chromatography with pulsed amperometric detection analysis

Thin layer chromatography (TLC) was performed on silica gel 60 F254, 20 × 20 cm TLC sheets (Merck, Darmstadt, Germany). The TLC plates were developed in *n*-butanol:acetic acid:water (2:1:1, v v^−1^) solvent system for 6 h. The carbohydrates were visualized with orcinol/sulfuric acid staining and compared with a simultaneous run of a mixture of glucose and MOS (DP2 to DP7).

Carbohydrate samples were diluted 3:100 in DMSO and analysed by HPAEC-PAD on an ICS3000 workstation (Thermo Scientific, Amsterdam, The Netherlands), equipped with a CarboPac PA-1 column (Thermo Scientific; 250 × 2 mm) and an ICS3000 electrochemical detection module. A gradient of sodium acetate from 10 to 240 mM in 100 mM NaOH was applied over 57 min at 0.25 ml min^−1^ flow rate. The injection volume of each sample was 5 μl. The identity of the peaks was assigned using commercial oligosaccharide standards.

### HPSEC analysis

HPSEC analyses of the product mixtures were performed using a size exclusion chromatography system (Agilent Technologies 1260 Infinity) equipped with a multi angle laser light scattering detector (SLD 7000 PSS, Mainz), a viscometer (ETA-2010 PSS, Mainz) and a differential refractive index detector (G1362A 1260 RID Agilent Technologies), as described before^26^. Separation was carried out by using three PFG-SEC columns with porosities of 100, 300 and 4000 Å, coupled with a PFG guard column. DMSO-LiBr (0.05 M) was used as eluent at a flow rate of 0.5 ml min^−1^. The system was calibrated and validated using a standard pullulan kit (PSS, Mainz, Germany) with *M*_w_ ranging from 342 to 805 000 Da. The specific RI increment value dn/dc was also measured by PSS and was 0.072 ml g^−1^ (private communication with PSS). The multiangle laser light scattering signal was used to determine the molecular masses of the amylose V and the polymer generated by the *L. fermentum* GtfB-ΔN enzyme. The specific RI increment value, dn/dc for these polysaccharides in this system was taken to be the same as for pullulan. The molecular mass of the *L. reuteri* GtfB-ΔN polymer was determined by universal calibration method. WinGPC Unity software (PSS, Mainz) was used for data processing. Measurements were performed in duplicate.

### Synthesis, isolation and characterization of the products synthesized by *L. fermentum* NCC 2970 GtfB from amylose

Purified GtfB-ΔN (0.25 mg) was incubated with amylose V for 48 h at 37 °C under the conditions described above in “Substrate utilization by *L. fermentum* NCC 2970 GtfB”. The product mixture obtained was fractionated by size-exclusion chromatography on a BioGel P2 column (2.5 × 50 cm; Bio-Rad, Veenendaal, The Netherlands) using 10 mM NH_4_HCO_3_ as eluent at a flow rate of 48 ml h^−1^. The poly-/oligo-saccharides present in the different Biogel P2 pools were analyzed by matrix-assisted laser-desorption ionization time-of-flight mass spectrometry (MALDI-TOF-MS), nuclear magnetic resonance (NMR) spectroscopy and methylation analysis. The polysaccharide was also subjected to Smith degradation analysis.

### MALDI-TOF mass spectrometry

MALDI-TOF-MS measurements were recorded on an Axima™ mass spectrometer (Shimadzu Kratos Inc., Manchester, UK) equipped with a nitrogen laser (337 nm, 3 ns pulse width). Sample solutions (1 μl) were spotted on a MALDI target and mixed immediately with 1 μl 10 mg ml^−1^ 2,5-dihydroxybenzoic acid matrix solution in 40% aqueous acetonitrile. Positive-ion mode spectra were recorded using the reflector mode at a resolution of 5000 Full Width at Half Maximum (FWHM) and delayed extraction optimised at 800 *m*/*z* by software control. Mass spectra were generally acquired from 1 to 5000 *m*/*z* with ion-gate blanking at 200 *m*/*z*.

### NMR spectroscopy

One- and two- dimensional ^1^H nuclear magnetic resonance (NMR) spectra were recorded on a Varian Inova 500 spectrometer (NMR Center, University of Groningen), using D_2_O as solvent and at a probe temperature of 298 K. Prior to analysis, samples were exchanged twice in D_2_O (99.9 atom% D, Cambridge Isotope Laboratories, Inc., Andover, MA) with intermediate lyophilization, and then dissolved in 0.6 ml of D_2_O. One-dimensional 500-*MHz*^1^H NMR spectra were recorded at a 4 000 *Hz* spectral width and 16k complex points, using a WET1D pulse to suppress the HOD signal. Two-dimensional ^1^H–^1^H spectra (COSY, TOCSY MLEV17 30, 50, and 150 ms, and ROESY 300 ms) were recorded with 4 000 *Hz* spectral width, collecting 200 increments. In case of TOCSY spectra 2 000 complex data points were collected, for COSY and ROESY spectra 4000 complex data points were used. 2D ^13^C-^1^H NMR spectra were recorded in 128 increments of 2000 complex points with 4000 *Hz* spectral width in *t2* and 10 000 *Hz* in *t1*. All NMR spectra were processed with MestReNova 5.3 (Mestrelabs Research SL, Santiago de Compostella, Spain). Manual phase correction was performed and a Whittacker smoother baseline correction was applied. Chemical shifts (δ) were expressed in ppm and calibrated with the internal standard acetone (δ 2.225 ppm for ^1^H and δ 31.08 ppm for ^13^C). The percentage of different linkages was estimated by integration of the respective signal peak areas.

### Methylation analysis

Methylation analysis was performed as described previously^36^. Briefly, the polymer and oligosaccharides samples (~5 mg) were per-methylated using CH_3_I and solid NaOH in DMSO, and subsequently hydrolyzed with trifluoroacetic acid. Partially methylated monosaccharides were reduced with NaBD_4_. The resulting partially methylated alditols were per-acetylated using pyridine:acetic anhydride (1:1 v/v) at 120 °C yielding mixtures of partially-methylated alditol acetates (PMAAs). PMAAs were analysed by GLC-EI-MS and GLC-FID as described^36^.

### Smith degradation

Samples of 1–2 mg polysaccharide were dissolved in 1 ml 50 mM NaIO_4_ in 100 mM NaOAc (pH 4.1) and stirred for 112 h at 4 °C in the dark. Excess IO_4_^−^ was neutralised by adding 300 μL ethyleneglycol. The degraded product was dialyzed in a SpectraPor 1000 Da cut-off dialysis floater against running tap water for 48 h. After dialysis the sample was reduced with NaBH_4_ at room temperature overnight, followed by dialysis as described above. The reduced polysaccharide sample was lyophilized and hydrolyzed in 1 ml 90% formic acid at 90 °C for 30 min. After cooling to room temperature formic acid was evaporated by N_2_ stream. The dried polysaccharide fragments were analyzed as TMS derivatives on GLC-EI-MS and GLC-FID, and by HPAEC-PAD as described previously.

### *L. fermentum* NCC 2970 GtfB product analysis with hydrolytic enzymes

The polymer produced by GtfB-ΔN was dissolved at a concentration of 5 mg ml^−1^ in 50 mM sodium acetate buffer pH 5.0, and incubated separately with an excess of α-amylase (*Aspergillus oryzae*; Megazyme), dextranase (*Chaetomium erraticum*; Sigma-Aldrich), and pullulanase M1 (*Klebsiella planticola*; Megazyme) for 48 h at 37 °C. Starch, *L. reuteri* 121 GtfB IMMP polymer and *A. chroococcum* reuteran-like GtfD polymer, were used as positive controls for the α-amylase, dextranase and pullulanase treatments, respectively, resulting in complete degradation under these conditions. The degree of hydrolysis was examined by TLC analysis.

### Structural modeling of the *L. fermentum* NCC 2970 GtfB protein

The Phyre server^37^ was used to obtain a homology model for the *L. fermentum* NCC 2970 GtfB protein. Querying the GtfB sequence against the PDB database suggested that the structure of DSR-E ΔN_123_-GBD-CD2 from *L. mesenteroides* NRRL B-1299 DSRE (PDB: 3TTO^12^) is the highest scoring structure with the highest number of residues aligned (residues 537–1500, covering domains A, B, C, IV and V). However, since we wanted to focus on the active site at the interface of domains A and B, we chose the structure of *L. reuteri* 180 Gtf180-ΔN (PDB: 3KLK^20^), which has the highest homology with *L. fermentum* GtfB in these domains, for the Phyre one-to-one threading modeling protocol. The resulting model contained residues 674–1593 (40% homology to Gtf180-ΔN). The model was superposed with complex of *L. reuteri* 180 Gtf180-ΔN with maltose (PDB: 3KLL^20^), for acceptor substrate modelling.

## Results and Discussion

### *L. fermentum* NCC 2970 carbohydrate-active enzymes

The genome sequences of more than 2500 isolates from the Nestlé Culture Collection have been subjected to analysis with a focus on the discovery and characterization of novel GH70 enzymes. The *L. fermentum* NCC 2970 genome was found to encode a total of 32 putative carbohydrate-active enzymes (CAZymes), as analyzed using dbCAN (Table S1). Of these, a putative GH70 family member (CDS0221) annotated as a dextransucrase (EC 2.4.1.5) was found located next to a transposase suggesting the potential acquisition of this gene via horizontal gene transfer. Interestingly, the *L. fermentum* NCC 2970 genome also encodes CAZymes enabling degradation of prebiotic compounds, i.e. compounds that are not digested by human enzymes^38^,^39^,^40^. These are (1) a member of the GH13 family (CDS1589), annotated as a α-glucosidase, putatively involved in isomalto-oligosaccharide degradation; (2) a member of the GH32 family (CDS0603) annotated as a β-fructofuranosidase, putatively involved in degradation of fructo-oligosaccharides; (3) 3 different GH2 family members annotated as β-galactosidases (CDS0592, CDS1127, CDS1128), putatively involved in lactose, galacto-oligosaccharide or galactan degradation. Furthermore, a cluster of 5 genes was shown to be relatively unique to *L. fermentum* NCC 2970 and contained three proteins without any function attributed (CDS0595, CDS0596 and CDS0599) as well as two glycoside hydrolases: a member of the GH3 family (CDS0597) annotated as a β-N-acetylhexosaminidase (EC 3.2.1.52), and a GH78 family member (CDS0598) annotated as an α-L-rhamnosidase (EC 3.2.1.40). Interestingly, adjacent to this cluster, also a putative xylan 1,3-β-xylosidase (EC 3.2.1.72) belonging to the GH43 family (CDS594) was found. Overall this region of the *L. fermentum* NCC 2970 genome may be involved in the degradation of plant or fungi derived substrates as enzymes present were previously shown to hydrolyze chitin derived substrates^41^, flavonoid rhamnoglycosides (i.e. nagirin, rutin and hesperidin)^42^ or β-1,3-xylan^43^.

### *L. fermentum* NCC 2970 GtfB protein sequence analysis

**Figure 1:**
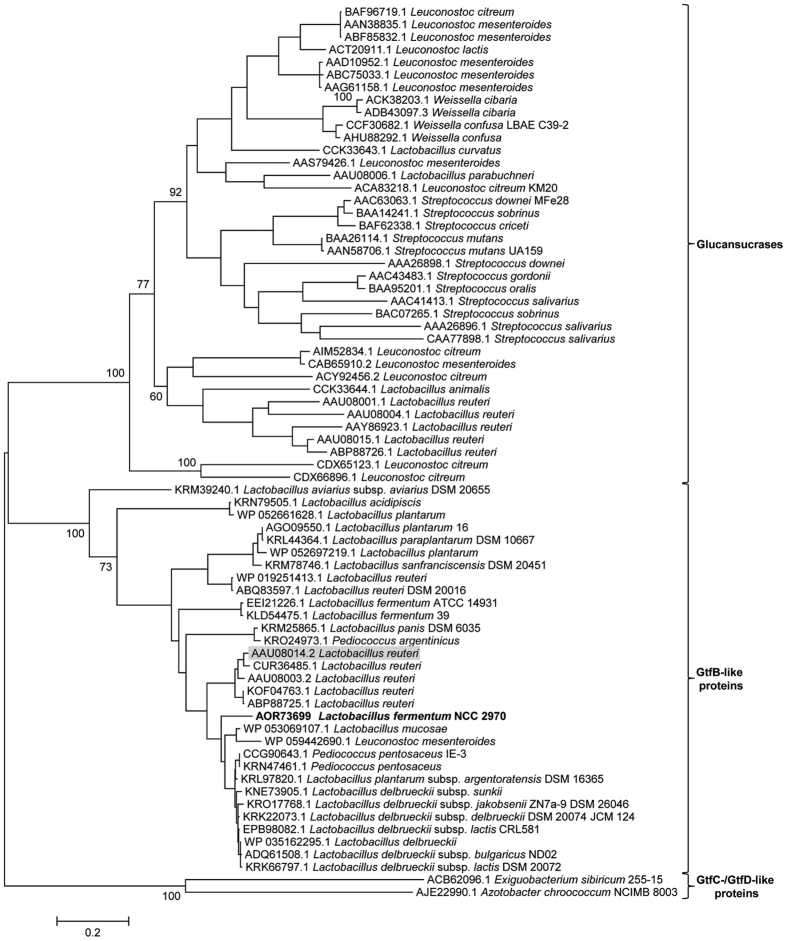
Phylogenetic tree constructed on the basis of complete amino acid sequence alignment of some of the characterized GH70 proteins annotated in the CAZy database, and of (putative) GH70 GtfB-like proteins identified by a BLAST search using the *L. fermentum* NCC 2970 GH70 protein as query sequence (shown in bold). The evolutionary history was inferred by using the Maximum Likelihood method based on the JTT matrix-based model. The bar represents a genetic distance of 0.2 substitutions per position (20% amino acid sequence difference). The bootstrap values adjacent to the main nodes represent the probabilities based on 1000 replicates. The protein sequences are annotated by their Genbank accession number and bacterial origin. The *L. reuteri* 121 GtfB 4,6-α-GTase is highlighted with a grey background.

Annotation of the *L. fermentum* NCC 2970 genome thus resulted in identification of a single GH70 family protein. BLASTp analysis of this *L. fermentum* NCC 2970 GH70 protein revealed that the closest homologs of this protein all were members of the GtfB-like 4,6-α-GTase GH70 subfamily with more than 47% identical amino acid sequences, including 3 further *L. fermentum* strains, namely 39, ATCC 14931 and 28-3-CHN (See supplementary Table S2). The phylogenetic relationship of the *L. fermentum* NCC 2970 GH70 protein to other characterized GH70 enzymes and (putative) GH70 4,6-α-GTases identified by this BLASTp search is depicted in Fig. 1. Glucansucrases (GSs) are present in the genomes of lactic acid bacteria of the genera *Leuconostoc, Streptococcus, Lactobacillus, Oenococcus* and *Weissella*, but most genes encoding putative GtfB homologs are currently found in *Lactobacillus* strains (except 4 GtfB-like proteins present in *Pediococcus* and *Leuconostoc* strains). The *L. fermentum* NCC 2970 GH70 enzyme clustered together with the *L. reuteri* 121 GtfB 4,6-α-GTase and its homologs, indicating that this protein is a member of the GtfB-type of GH70 subfamily. The GtfB-like proteins display a domain organization resembling that of GH70 GSs with a circularly permuted (β/α)_8_ barrel^24^, however, biochemically they are more related to the *E. sibiricum* 255-15 GtfC and *A. chroococcum* NCIMB 8003 GtfD enzymes^23^,^26^. In agreement with these observations, the GtfB-like GH70 subfamily is evolutionary more closely related to GSs, but it has an intermediate position between the GSs and the GtfC and GtfD 4,6-α-GTases encoded by *E. sibiricum* 255-15 and *A. chroococccum* NCIMB 8003, respectively.

The newly identified GtfB gene sequence from *L. fermentum* NCC 2970 encodes a polypeptide consisting of 1593 amino acids with a calculated molecular mass of 180 kDa. In agreement with the extracellular location of GH70 enzymes, this *L. fermentum* GtfB was predicted to function as an extracellular protein by the CELLO program. Analysis of the N-terminus of the amino acid sequence of *L. fermentum* NCC 2970 GtfB revealed that this protein contains the classical Gram-positive N-terminal signal peptide of 39 amino acids. The *L. fermentum* NCC 2970 GtfB shares significant amino acid identity (77% identity, 57% coverage) with the *L. reuteri* 121 GtfB, the first characterized GH70 member with 4,6-α-glucanotransferase activity^24^. The *L. fermentum* 39, ATCC14931 and 28-3-CHN GtfB proteins are 99% identical to each other, but only 79%, 78% and 73% with the NCC 2970 GtfB protein.

**Figure 2:**
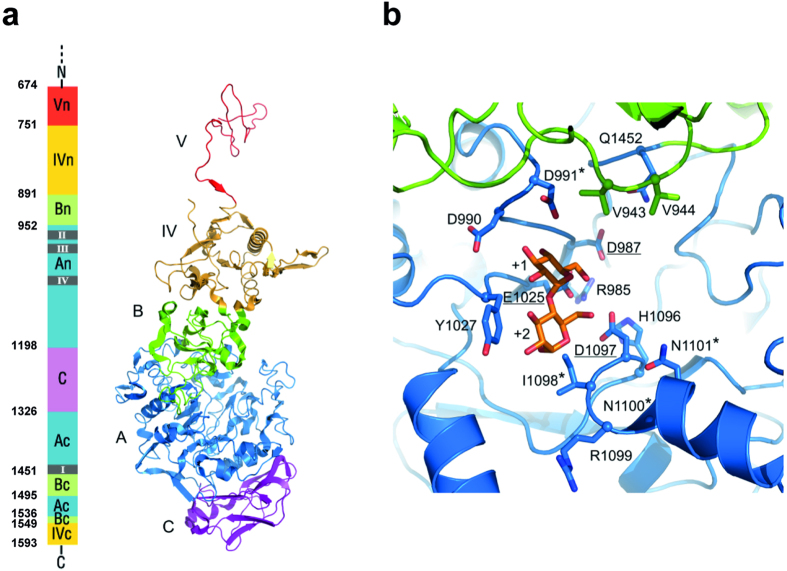
Homology model for *L. fermentum* NCC 2970 GtfB. Tertiary structure prediction was accomplished by using the Phyre2 server^37^. Among the different templates found, the *L. reuteri* 180 Gtf180-ΔN (PDB: 3KLK^20^), was chosen as it displayed the highest homology with *L. fermentum* NCC 2970 GtfB in domains A and B. (**a**) Schematic representation of the *L. fermentum* GtfB 3D model structure. Domains A, B, C, IV and V are colored in blue, green, magenta, yellow and red, respectively. Only part of the N-terminal half of domain V is present in the homology model. (**b**) Close-up of the active site at the interface of domains A (blue) and B (green). The catalytic residues (underlined) and other residues lining the active site, present in motifs I-IV are shown in stick representation. Residues that are different from previously identified 4,6-α-GTases are indicated with an asterisk. A maltose acceptor substrate bound in subsites +1 and +2 (orange carbon atoms) is shown based on the Gtf180/maltose complex (PDB: 3KLL^20^).

The three-dimensional (3D) model structure of the *L. fermentum* NCC 2970 GtfB protein was deduced by homology modeling using *L. reuteri* 180 Gtf180-ΔN GS as the template (Fig. 2). This homology model contained residues 674–1593, and revealed that the *L. fermentum* GtfB displays the typical U-fold domain organization characteristic of most GH70 proteins consisting of five domains (A, B, C, IV and V), with a circularly permuted catalytic (β/α)_8_ barrel (Fig. 2a). Similar to *L. reuteri* 121 GtfB^27^, domains A, B, C and IV of the *L. fermentum* NCC 2970 GtfB protein are built up from discontinuous N- and C-terminal stretches of the polypeptide chain. Notably, sequence alignment predicted that *L. fermentum* GtfB has no C-terminal domain V segment; while only part of domain V (residues 674–750) is present in the homology model obtained from one-to-one threading with *L. reuteri* 180 Gtf180-ΔN. It is likely that other N-terminal residues further complete this domain. Indeed, a homology model based on the DSR-E ΔN_123_ GBD-CD2 structure contained a complete domain V (residues 537–750) (not shown). Besides, the *L. fermentum* NCC 2970 GtfB has a variable N-terminal domain of ~500 residues present in many glucansucrases and GtfB-like proteins of *Lactobacillus* species that is believed to be involved in cell wall attachment^44^.

**Figure 3:**
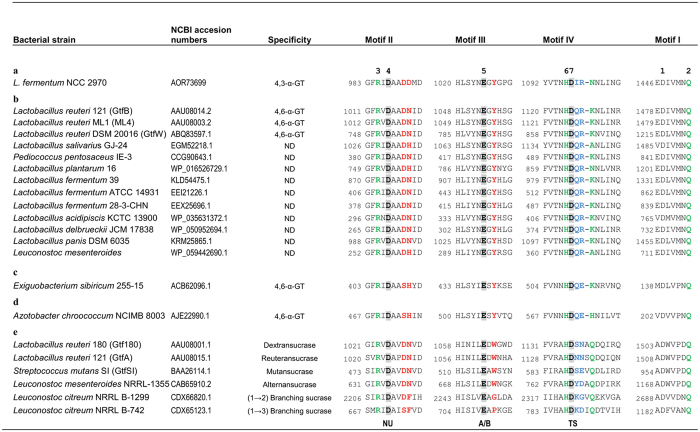
Sequence alignment of conserved motifs I-IV in the catalytic domains of the *L. fermentum* NCC 2970 4,3-α-GTase GtfB enzyme (**a**), (putative) GtfB-like 4,6-α-GTase enzymes (**b**), *E. sibiricum* GtfC 4,6-α-GTase enzyme (**c**), *A. chroococcum* GtfD 4,6-α-GTase enzyme (**d**), and glucansucrase enzymes (**e**). The seven strictly conserved amino acid residues in GH70 enzymes (indicated by the numbers 1 to 7 above the sequences) are also conserved in the novel *L. fermentum* 4,3-α-GT GtfB protein. Amino acids that constitute the catalytic triad are shown in bold and lightly shaded. Residues forming acceptor substrate binding subsites −1, +1 and +2 in glucansucrase Gtf180-ΔN^20^ are highlighted in green, red and blue, respectively. Symbols: NU = nucleophile, A/B = general acid/base, TS = transition state stabilizer.

Aiming to predict the reaction and/or product specificity of the *L. fermentum* NCC 2970 GtfB enzyme, the homology regions I-IV of this protein were identified by sequence alignments with other GH70 family proteins (Fig. 3) and further inspected in the Gtf180-ΔN-based homology model (Fig. 2b). These four homology motifs contain the catalytic and substrate-binding residues, and thus are regarded as sequence fingerprints for the individual enzyme specificities in both GH70 and GH13 family enzymes^15^,^45^. The order of the conserved regions I-IV in the *L. fermentum* NCC 2970 GtfB is II-III-IV-I and corresponds to the order found in GSs and GtfB-like 4,6-α-glucanotransferases, reflecting its circularly permuted domain organization. The seven residues strictly conserved in motifs I-IV of GH70 family enzymes^45^ are present in the *L. fermentum* GtfB. Among these seven residues, the three putative catalytic residues Asp987, Glu1025 and Asp1097 (*L. fermentum* NCC 2970 GtfB numbering), were identified as the nucleophile, the general acid/base catalyst and the transition state stabilizer, respectively. The arrangement of the catalytic residues is identical to that in known GS structures, but other residues lining the active site show differences. For example, similar to other GtfB-like enzymes, a Tyr residue in motif III replaces the Trp residue (W1065 in *L. reuteri* Gtf180 GS) that is conserved in all GSs, in which it provides an aromatic stacking interaction at subsites +1/+2 with acceptor substrates^20^,^22^; in branching sucrases the tryptophan is absent^12^,^13^. It is noteworthy that *E. sibiricum* GtfC and *A. chroococcum* GtfD 4,6-α-GTase enzymes also have a Tyr at this position, which therefore represents one the main differences discriminating a GH70 enzyme active on starch/maltodextrin substrates from “classical” GSs. A second difference is Asp991 of *L. fermentum* NCC 2970 GtfB; it replaces the conserved Asn present in GSs and most GtfB-like 4,6-α-GTases. In the GS of *L. reuteri* 180 this residue was found to be essential for activity and linkage specificity^31^. The preceding Asp is conserved in *L. fermentum* GtfB (Asp 990). Finally, residues in motif IV were previously shown to be involved in determining linkage specificity; in particular the first and fourth residue downstream the transition state stabilizer^20^,^29^,^46^,^47^. Glucansucrases show variation in this region, while GtfB-like 4,6-α-GTases have an almost completely conserved QRKN motif (note that the alignment predicts a one-residue gap, Fig. 3). The *L. fermentum* NCC 2970 GtfB instead has IRNN, also different from the GtfC and GtfD enzymes. Thus, despite the fact that the *L. fermentum* NCC 2970 GtfB exhibits clear sequence similarity with the *L. reuteri* 121 GtfB 4,6-α-GTase protein, it contains unique sequence features. The homology regions I-IV of the GtfB proteins of *L. fermentum* strains 39, ATCC 14931 and 28-3-CHN are similar to that of the *L. reuteri* 121 GtfB (Fig. 3). Inspection of the *L. fermentum* NCC 2970 GtfB homology model and the likely binding mode of an acceptor substrate (maltose) confirmed that the residues mentioned above indeed cluster around the active site and substrate-binding regions (Fig. 2b), and that the residues in *L. fermentum* NCC 2970 GtfB that are different from previously identified 4,6-α-GTases likely are in functionally important positions for determining the substrate- and product specificity of the enzyme. Therefore, we investigated the *L. fermentum* NCC 2970 GtfB substrate and product specificity in detail.

### Purification and biochemical characterization of the *L. fermentum* NCC 2970 GtfB

The *L. fermentum* NCC 2970 GtfB-ΔN protein was successfully expressed in *E. coli* BL21(DE3) Star. Under the growth and inductions conditions used, the expression level of GtfB-ΔN was relatively low in both the soluble and insoluble fractions (Supplementary Fig. S1). The enzyme was purified from the soluble fraction by two chromatographic steps consisting of metal chelate chromatography and anion-exchange chromatography, as described in the Experimental Section. SDS-PAGE analysis of the purified enzyme revealed a single protein band with an apparent molecular mass of ~110 kDa (Supplementary Fig. S1) which fits the theoretical value deduced from the sequence. As a result of this purification process, a total of 0.4 mg of pure GtfB-ΔN protein per L of *E. coli* culture was obtained. A domain V truncated variant (with amino acids 751 to 1593) was constructed with the aim of increasing protein expression. This truncation approach did not significantly improve the expression level of the *L. fermentum* NCC 2970 GtfB protein (Data not shown).

The effects of pH and temperature on the enzyme activity were determined by the iodine-staining assay using amylose V as substrate. The purified recombinant *L. fermentum* NCC 2970 GtfB exhibited the highest activity at pH 5.5 and retained more than 80% of this activity over a pH from 4 to 6 (Supplementary Fig. S2a). The enzyme was active between 30 and 60 °C, showing its maximal activity at 50 °C, but the activity decreased drastically when the reaction was performed at 65 °C (Supplementary Fig. S2b). In addition, the enzyme showed low stability at temperatures above 45 °C in 20 mM Tris-HCl buffer (pH 8.0) (Supplementary Fig. S2c). Similar pH and temperature optimum values were reported for the *L. reuteri* 121 GtfB 4,6-α-GTase enzyme^27^. The specific total activity of the purified *L. fermentum* NCC 2970 GtfB enzyme on 0.125% (w v^−1^) amylose V in sodium acetate buffer (pH 5.5), containing 1 mM CaCl_2_ was determined using an iodine-staining assay to be 22 ± 0.36 U mg^−1^ of protein. This value is around 10 times higher than the one reported for the GtfB from *L. reuteri* 121 (under its optimal conditions of pH 5.0 and 40 °C), namely, 2.8 U mg^−1^ 27. The activity of the *L. fermentum* NCC 2970 GtfB was also assayed in the presence of the chelating agent EDTA at a final concentration of 10 mM, resulting in a slight (20%) inhibition.

### Substrate and product specificity of the *L. fermentum* NCC 2970 GtfB

**Figure 4:**
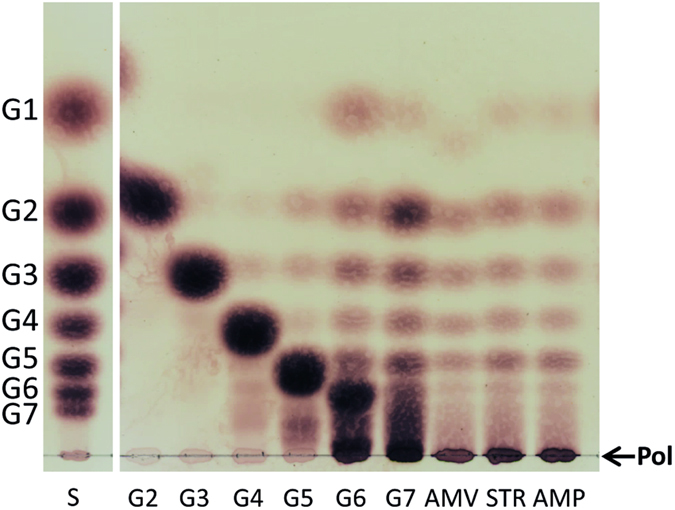
TLC analysis of the product mixtures synthesized by the *L. fermentum* NCC 2970 GtfB enzyme in incubations with malto-oligosaccharides (DP2-DP7), amylose V, starch, and amylopectin. The reaction mixtures containing 25 mM malto-oligosaccharides or 0.6% (w/v^−1^) polysaccharides and 25 μg ml^−1^ of enzyme were incubated at 37 °C and pH 5.5 during 24 h. S, standard; G1, glucose; G2-G7, maltose to maltoheptaose; AMV, amylose V; STR, starch; AMP, amylopectin; Pol, polymer.

The differences observed in the homology motifs suggested that the *L. fermentum* NCC 2970 GtfB may possess a new enzymatic reaction and/or product specificity. Therefore the *L. fermentum* GtfB enzyme was incubated with different oligosaccharides and polysaccharides at 37 °C for 24 h, and the reaction products were analysed by TLC (Fig. 4). Similar to *L. reuteri* GtfB, the *L. fermentum* NCC 2970 GtfB enzyme failed to act on sucrose, panose, nigerose, and isomalto-oligosaccharides with DP2, DP3, and DP5 (Data not shown). Instead, the *L. fermentum* GtfB enzyme showed both hydrolysis and transglycosylase (disproportionation) activity on malto-oligosaccharides (MOS) of DP 6 and 7, evident from the accumulation of products with lower- and higher-molecular-mass than the MOS substrates. *L. fermentum* GtfB was not active on MOS of DP below 4 and showed low disproportionating activity with maltopentaose (DP5); for *L. reuteri* 121 GtfB activity was already observed with maltotriose (DP3)^23^. Incubation of amylose V, potato starch and amylopectin with the *L. fermentum* NCC 2970 GtfB enzyme resulted in the appearance of a range of lower molecular mass products indicating that the enzyme was also active on these polymers. It should be noted that the *L. fermentum* GtfB enzyme produced significantly larger amounts of oligosaccharide products than the *L. reuteri* 121 GtfB, which mainly synthesized a (modified) polymer from amylose V^23^. As observed by TLC, MOS of DP 2 to 5 accumulated from the various polymeric substrates, reflecting that *L. fermentum* GtfB requires maltohexaose or longer MOS as glucose donor substrates.

**Figure 5:**
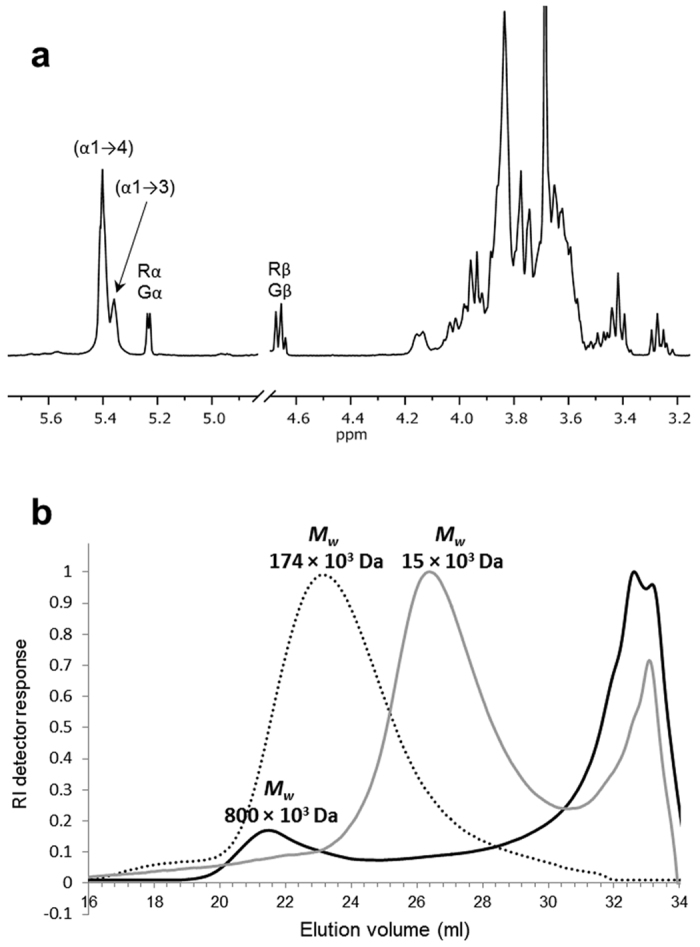
Structural analysis of the products synthesized by the incubation of 0.6% (w v^−1^) amylose V with 25 μg ml^−1^ of *L. fermentum* NCC 2970 GtfB for 24 h at 37 °C and pH 5.5. (**a**) ^1^H NMR spectra (D_2_O, 300 K) of the product mixtures formed. The anomeric signals indicated as Gα/β and Rα/β correspond to free glucose and reducing -(1 → 4)-D-Glc*p* units, respectively. Chemical shifts are given in parts per million relative to the signal of internal acetone (δ 2.225). (**b**) HPSEC molecular mass distribution of the reaction products generated from amylose V by the *L. fermentum* NCC 2970 GtfB enzyme. The elution profiles of the starting amylose V substrate (dashed line) and of the products synthesized by *L. reuteri* 121 GtfB from amylose V (grey line) are also included.

Aiming to study the product specificity of the *L. fermentum* NCC 2970 GtfB, the maltoheptaose and amylose V derived product mixtures were analyzed by one-dimensional ^1^H NMR spectroscopy. As an example, the ^1^H-NMR spectrum of the product mixture generated from amylose V is depicted in Fig 5a. ^1^H-NMR analysis of both product mixtures revealed the presence of two groups of anomeric overlapping signals at δ ~5.40 and 5.35 corresponding to (α1 → 4) linkages and newly synthesized (α1 → 3) linkages. The presence of (α1 → 3) linkages was corroborated by the structural-reporter-group signal for (α1 → 3) linkages at δ_H-5_ 4.16 ppm^46^. The spectra also showed signals corresponding to free glucose units (Gα H-1, δ 5.225; Gβ H-1, δ 4.637) and 4-substituted reducing-end glucose residues (Rα H-1, δ 5.225; Rβ H-1, δ 4.652). Small signals corresponding to trace amounts (less than 1%) of (α1 → 6) linkages (H-1, δ~4.97) were detected as well. The molar ratios of the (α1 → 4)-linked, (α1 → 3)-linked glucose residues for maltoheptaose and amylose V products were 86:14 and 81:19, respectively. Methylation analysis of the product mixture generated by the *L. fermentum* NCC 2970 GtfB from amylose V showed the presence of terminal, 3-substituted, 4-substituted, and 3,4-disubstituted glucopyranose residues in a molar percentage of 25, 16, 55, and 4%, which is in agreement with the linkage ratios determined by ^1^H NMR and confirms that the *L. fermentum* GtfB exhibits (α1 → 3) linkage specificity. In contrast to the *L. reuteri* GtfB enzyme that only generates linear (α1 → 6) glucan chains (4,6-α-GTase), the *L. fermentum* NCC 2970 GtfB acts as a 4,3-α-glucanotransferase (4,3-α-GTase) catalyzing the cleavage of (α1 → 4) linkages and the formation of new (α1 → 3) in linear or branched orientations. The high relative amount of terminal glucopyranose residues (25%) detected in the reaction product mixture correlates with the presence of glucose and small MOS obtained as side products of the *L. fermentum* GtfB hydrolase/transglycosylase activity.

The action of the *L. fermentum* NCC 2970 GtfB enzyme on amylose was also characterized by HPSEC with multidetection (Fig. 5b). The HPSEC profile of the starting amylose V substrate exhibited a single peak eluting at 23.0 ml, with an average *M*_w_ of 174 × 10^3^ Da, whereas *L. fermentum* GtfB-treated amylose V showed two different molecular mass distribution peaks: An early peak eluting at 21.5 ml corresponding to a high molecular mass polymer with an average *M*_w_ of 800 × 10^3^ Da and, a second peak corresponding to oligosaccharides with an average *M*_w_ of 1400 Da. In case of the *L. reuteri* 121 GtfB 4,6 α-GT, the peak corresponding to the IMMP eluted at 26.5 ml and had an average *M*_w_ of 15 × 10^3^ Da. Thus, the *L. fermentum* GtfB is capable of synthesizing a polymer with a *M*_w_ value about 4 and 50 times higher than those of the starting amylose V substrate and the IMMP product, respectively. However, based on the refractive index signal this polymer represents less than 20% of the total product mixture of the *L. fermentum* GtfB, while a significant proportion of low molecular mass glucans are present.

### Structural characterization of the products synthesized by the *L. fermentum* NCC 2970 GtfB from amylose V

For a more detailed structural characterization, the products produced by the *L. fermentum* NCC 2970 GtfB from amylose were purified by size-exclusion chromatography on Biogel P2. The seven fractions obtained, designated as **F1-F7**, were analysed separately by MALDI-TOF MS and ^1^H NMR spectroscopy (Table 1). Fractions **F6** and **F7** contained the hydrolysis products maltotriose (DP3) and maltose (DP2), respectively, and were not further studied. The 1D ^1^H NMR spectrum (Fig. 5a) showed α-anomeric signals at δ 5.41 and 5.37; this region may contain anomeric signals of both (α1 → 4) and (α1 → 3) linked α-D-Glc*p* residues^48^. Therefore, fractions **F1-F5** were also subjected to methylation analysis in order to determine the presence of (α1 → 4) and (α1 → 3) linkages and to determine the degree of branching. The methylation analysis data fit with the peak at δ 5.37 corresponding to (α1 → 3)-linked residues (Table 1). The ^1^H NMR and methylation analysis of the Biogel P2 fractions demonstrated that the percentage of (α1 → 3) linkages increased with increasing DP of the products (Table 1). The highest molecular mass product **F1** containing polymeric material of DP >30 showed an increased percentage of  → 3)-Glc*p*-(1 →  glucosyl units (28%) and branching  → 3,4)-Glc*p*-(1 →  glucosyl units (8%) over those in the total reaction product mixture, 16 and 4%, respectively.

**Figure 6:**
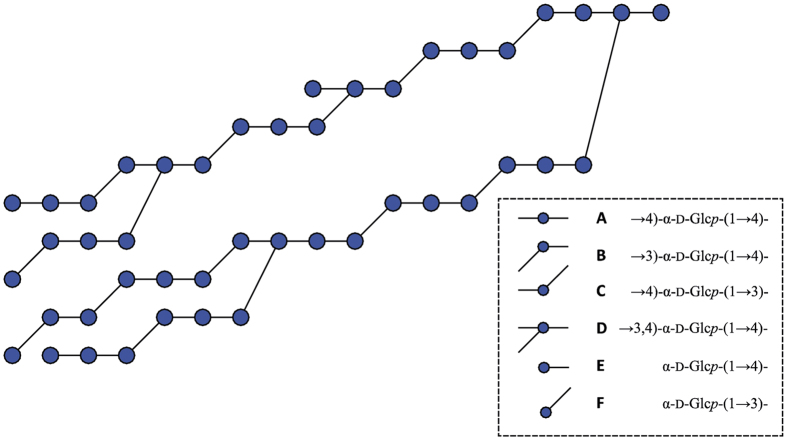
Composite model of the *L. fermentum* NCC 2970 GtfB polysaccharide product. The composite model includes all structural elements identified by 1D and 2D NMR spectroscopy analysis, methylation analysis and Smith degradation analysis. Residue labels (**A**–**F**) presented on the right correspond with those in Table S3.

To unravel the structural elements present in fraction **F1**, 2D NMR (^13^C-^1^H HSQC, ^1^H-^1^H TOCSY, and ^1^H-^1^H ROESY) experiments were performed (Supplementary Fig. S3). For a detailed description of the structural characterization of fraction **F1**, see supplementary information. Most notably, the 2D NMR data fit with the occurrence of alternating (α1 → 3)/(α1 → 4) linkages and (α1 → 3,4) branching points in the *L. fermentum* NCC 2970 GtfB polymer, whereas signals corresponding to sequential (α1 → 3)-linkages were not identified. The absence of consecutive (α1 → 3)-linkages in fraction **F1** was further confirmed by Smith degradation analysis (Supplementary Fig. S4). Taking together all data from 1D and 2D NMR spectroscopy analysis, methylation analysis and Smith degradation analysis, a composite model for **F1** was constructed, showing all identified structural elements in the correct relative abundance (Fig. 6).

### Oligosaccharides formed in time from maltoheptaose and amylose V by the *L. fermentum* NCC 2970 GtfB enzyme

**Figure 7:**
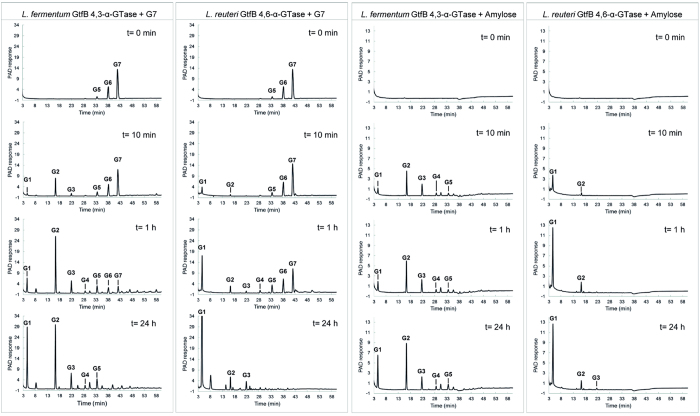
HPAEC-PAD profile of the oligosaccharide mixtures formed upon incubation of maltoheptaose and amylose V with 25 μg ml^−1^ of *L. fermentum* NCC 2970 GtfB and *L. reuteri* 121 GtfB for t = 10 min, 1 h, and 24 h (pH 5.5, 37 °C). The identity of peaks was assigned using commercial oligosaccharide standards. G1, glucose; G2–G7, maltose to maltoheptaose.

To gain a better understanding of the action pattern of the *L. fermentum* NCC 2970 GtfB enzyme, time course experiments were performed with the maltoheptaose and amylose V substrates. The oligosaccharide products formed after 10 min, 1 h and 24 h of reaction were analyzed by HPAEC. At the early stage of the reaction with maltoheptaose (slightly contaminated with G5 and G6), G2 was identified as the main reaction product (Fig. 7). Small peaks corresponding to glucose (G1) and maltotriose (G3) were also identified, whereas a peak with an unknown structure but with a high DP eluted at 58 min. The release of G2, together with the synthesis of a compound with a higher DP, suggests that the *L. fermentum* GtfB enzyme catalyzes the transfer of a maltopentaosyl unit to a MOS acceptor substrate. Later in time, the amounts of glucose and MOS with DP2 to 5 increased, whereas after 24 h G6 and G7 had become depleted. In addition, as a result of the *L. fermentum* GtfB disproportionating activity, peaks that did not fit the MOS retention times and probably corresponding to oligosaccharides containing (α1 → 3) linkages, were detected at shorter and longer retention times than that of the G7 starting substrate. Incubation with the amylose V yielded G2 as the first clear reaction product, together with G1, G3, G4, G5 (Fig. 7). These profiles significantly differed from the ones obtained with the *L. reuteri* 121 GtfB enzyme when both G7 and amylose V are used as substrates (Fig. 7). With both substrates the *L. reuteri* 121 GtfB releases glucose (instead of G2) as the main first product at the beginning of the reaction. The pronounced accumulation of MOS with DP2 to DP5 by the *L. fermentum* NCC 2970 GtfB was not seen for the *L. reuteri* 121 GtfB. These results suggest that the *L. fermentum* and *L. reuteri* 121 GtfB enzymes differ in their mechanism of polymerization. The novel *L. fermentum* GtfB enzyme preferentially catalyzes the transfer of MOS of low DP, showing a mode of action more resembling that of the *A. chroococcum* GtfD enzyme^26^. On the other hand, the *L. fermentum* GtfB 4,3-α-GT seems to require MOS of a certain minimum length (at least DP6) to act on and to produce an α-glucan with an (α1 → 3)/(α1 → 4) alternating structure and with (α1 → 3) branching points. Finally, the data indicate that the *L. fermentum* NCC 2970 GtfB 4,3-α-GT active site may present more than one donor substrate binding subsite, as observed for enzymes belonging to the evolutionary related GH13 and GH77 families^49^,^50^.

### Enzymatic treatment of the *L. fermentum* NCC 2970 GtfB product

**Figure 8:**
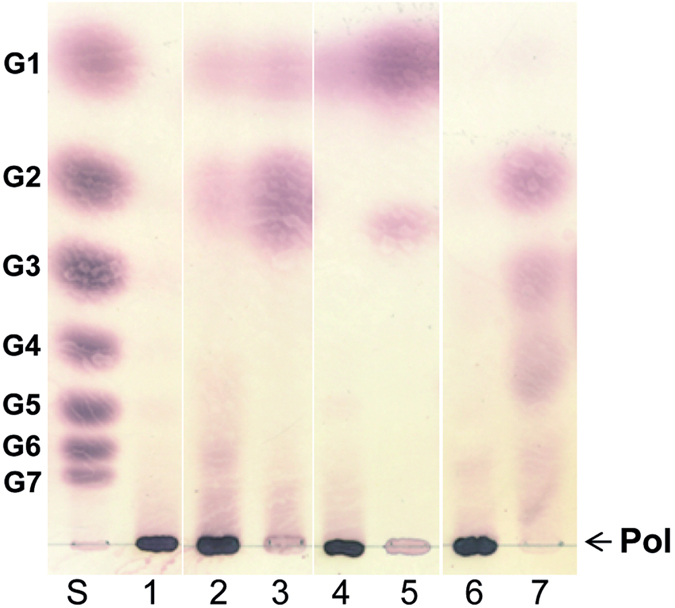
TLC analysis of the *L. fermentum* NCC 2970 GtfB polymer after treatment with *Aspergillus oryzae* α-amylase, *Chaetomium erraticum* dextranase and *Klebsiella planticola* pullulanase M1. Lane 1, *L. fermentum* GtfB polymer before the enzymatic treatments; Lanes 2–3, product mixtures generated by the α-amylase enzymatic treatment of the *L. fermentum* GtfB polymer and starch, respectively. Lanes 4–5, product mixtures generated by the dextranase enzymatic treatment of the *L. fermentum* GtfB polymer and IMMP, respectively. Lanes 6–7, product mixtures generated by the pullulanase enzymatic treatment of the *L. fermentum* GtfB polymer and the *A. chroococcum* GtfD polymer, respectively. Lane S, standard; G1, glucose; G2-G7, maltose to maltoheptaose; Pol, polymer.

To further characterize the polymer synthesized by the *L. fermentum* NCC 2970 GtfB enzyme (Biogel P2 fraction **F1**), this α-glucan was treated with a high activity dose of α-amylase, dextranase and pullulanase enzymes. The polymer produced by *L. fermentum* GtfB was resistant to the endo-(α1 → 4)-hydrolase activity of α-amylase. As revealed by TLC analysis, only trace amounts of glucose, maltose and high molecular mass oligosaccharides were detected after 48 h of α-amylase digestion (Fig. 8). Under the same reaction conditions, the starch control substrate was completely hydrolyzed, indicating that the presence of α-(1 → 3) linkages makes the *L. fermentum* GtfB polymer resistant to α-amylase digestion. The *L. fermentum* GtfB polymer was also subjected to dextranase and pullulanase enzymatic treatments. Dextranase catalyzes the endohydrolysis of consecutive (1 → 6) linkages in dextran, while pullulanase cleaves the (1 → 6) linkages present in pullulan, amylopectin and α- and β-limit dextrins of starch. Thus, for the dextranase and the pullulanase treatments, the polymers produced from amylose V by the *L. reuteri* GtfB and the *A. chroococcum* GtfD were included as positive controls, respectively. As expected, dextranase efficiently hydrolyzed the IMMP produced by the *L. reuteri* 121 GtfB, whereas pullulanase completely digested the reuteran-like polymer synthesized by the *A. chroococcum* GtfD enzyme. In contrast, no hydrolysis was observed in the case of the *L. fermentum* NCC 2970 GtfB polymer, which is in agreement with the absence of (1 → 6) linkages (less than 1%) deduced from the ^1^H NMR analysis.

## Conclusions

This paper describes the identification and biochemical characterization of the first GH70 enzyme cleaving (α1 → 4)-linkages and synthesizing (α1 → 3)-linkages, encoded by *L. fermentum* NCC 2970. We propose to name this enzyme (α1 → 4)-α-D-glucan: (α1 → 4), (α1 → 3)-α-D-glucan α-glucanotransferase, in short 4,3-α-glucanotransferase. Regarding its primary sequence, this protein clearly belongs to the novel GtfB-like GH70 subfamily which originally only comprised 4,6-α-glucanotransferases, however, it also possesses unique variations in residues forming the donor and acceptor binding subsites of GSs in particular in signature motifs II and IV. This suggests that the *L. fermentum* NCC 2970 GtfB active site displays distinctive features that may lead to its different specificity compared to other GtfB-like enzymes. Indeed, the *L. fermentum* NCC 2970 GtfB resembles previously characterized GtfB enzymes in using maltodextrins and starch as substrates, but instead of catalyzing the synthesis of consecutive (α1 → 6) linkages, it displays (α1 → 3) linkage specificity. The *L. fermentum* GtfB activity results in the synthesis of an unique α-glucan built up with different lengths of malto-oligosaccharides, interconnected by single (α1 → 3) linkages in linear and branched orientations.

Even though GSs have a wide product spectrum and synthesize various types of linkages, none of the GSs characterized so far produce an α-glucan consisting of (α1 → 4)- and (α1 → 3)-linkages. As far as we are aware, the structure of the *L. fermentum* NCC 2970 GtfB product also differs from that corresponding to α-glucans containing both (α1 → 4)- and (α1 → 3)-linkages produced by different lichens and fungi^51^,^52^. Indeed, most of these polysaccharides have linear structures^53^,^54^,^55^,^56^,^57^, whereas others are mainly composed of (α1 → 3)-linkages and/or do not present (α1 → 3,4)-branching points^58^,^59^. Owing to the novel structure of the polymer produced by *L. fermentum* NCC 2970 GtfB, it will be of interest to study the physico-chemical and functional properties of this α-glucan and to explore its applications. For example, the direct action of the *L. fermentum* GtfB on the starch present in food matrices is expected to result in the synthesis of slowly digestible starch derivatives. Overall, this study expands the diversity of the GH70 α-glucans that can be produced, and provides new insights in the understanding of the features that determine the reaction/product specificity of GH70 family proteins.

## Additional Information

**How to cite this article**: Gangoiti, J. *et al*. 4,3-α-Glucanotransferase, a novel reaction specificity in glycoside hydrolase family 70 and clan GH-H. *Sci. Rep.* **7**, 39761; doi: 10.1038/srep39761 (2017).

**Publisher's note:** Springer Nature remains neutral with regard to jurisdictional claims in published maps and institutional affiliations.

## Supplementary Material

Supplemental Information

## Figures and Tables

**Table 1:** Linkage composition of the fractions (F1-F7) yielded by size-exclusion chromatography on Biogel P2 of the product mixture obtained from the incubation of 0.6% (w v^−1^) amylose V with 25 μg ml^−1^ of *L. fermentum* NCC 2970 GtfB.

Sample	DP	Chemical shift (%)^a^			Methylation analysis (%)^b^		
		(α1 → 4)	(α1 → 3)	Glc*p* (1 →	→3)-Glc*p*-(1 →	→4)-Glc*p*-(1 →	→3,4)-Glc*p*-(1 →
F1	>30	60	40	6	28	58	8
F2	~8–20	71	29	12	21	61	6
F3	~6–8	80	20	14	15	66	5
F4	~5–6	88	12	21	10	67	2
F5	~4–5	89	11	21	10	64	5
F6	~3	100	0	ND	ND	ND	ND
F7	~2	100	0	ND	ND	ND	ND

^a^The data represent the ratios of integration of the peak areas of the (α1 → 4) linkage signal at 5.41 ppm and the (α1 → 3) linkage signal at 5.37 ppm in the ^1^H NMR spectra.

^b^The linkage distribution data are shown in molar percentages based on GLC intensities.
