# Supplementary material for: 4,3-α-Glucanotransferase, a novel reaction specificity in glycoside hydrolase family 70 and clan GH-H
Source: Sci Rep. 2017 Jan 6;7:39761. doi: 10.1038/srep39761 (PMC5216370; doi:10.1038/srep39761)
Supplement: Supplemental Information [file srep39761-s1.pdf]

Supplementary Information

4,3-α-Glucanotransferase, a novel reaction specificity in glycoside hydrolase family 70 and clan GH-H

Joana Gangoiti [a], Sander S. van Leeuwen [a], Gerrit J. Gerwig [a], Stéphane Duboux [b], Christina Vafiadi [b], Tjaard Pijning [c], Lubbert Dijkhuizen [a *]

[a] Microbial Physiology, Groningen Biomolecular Sciences and Biotechnology Institute (GBB), University of Groningen, Nijenborgh 7, 9747 AG Groningen, The Netherlands

[b] Nestlé Research Center, Vers-Chez-Les-Blanc, Lausanne, Switzerland

[c] Biophysical Chemistry, Groningen Biomolecular Sciences and Biotechnology Institute (GBB), University of Groningen, Nijenborgh 7, 9747 AG Groningen, The Netherlands

[*] Corresponding author

E-mail address: L.Dijkhuizen@rug.nl (L. Dijkhuizen)

SUPPLEMENTARY TEXT

SUPPLEMENTARY TABLES

SUPPLEMENTARY FIGURES

REFERENCES

SUPPLEMENTARY TEXT

Detailed structural characterization of the polymeric fraction F1

*1D 2D NMR analysis of the polymeric fraction **F1***

The 1D ^{1}H NMR spectrum (**Supplementary Fig. S3**) of the polysaccharide product (**F1**) showed sharp anomeric signals at δ 5.41 and δ 5.37 ppm, fitting the presence of (α1→4) and (α1→3) linked glucose units, respectively, in a 6:4 ratio [1, 2, 3], fitting with the methylation analysis data (**Table 2**).

Table S3 summarizes the NMR data. In the δ 5.41 anomeric track at least three overlapping types of residues were observed, as evident from the H2 signals in the 2D ^{1}H-^{1}H COSY spectrum (**Supplementary Fig. S3**, green) at δ 3.59, 3.63 and 3.69, respectively. Further analysis in the 2D TOCSY spectra with 50 ms (not shown) and 150 ms (**Supplementary Fig. S3**, grey) mixing time allowed for elucidation of three H-3 signals at δ 3.70, 3.85 and 3.97, and H-4 signals at δ 3.65-3.67 and 3.42, respectively. In case of a terminal α-D-Glc*p*-(1→4)- residue H-2, H-3 and H-4 signals are expected at δ 3.57, 3.71 and 3.42, respectively [3], for a (1→4)-α-D-Glc*p*-(1→4)- residue H-2, H-3 and H-4 are expected at δ 3.63, 3.96 and 3.65, respectively [3], whereas a (1→3)-α-D-Glc*p*-(1→4)- residue should render H-2, H-3 and H-4 at δ 3.68, 3.85 and 3.65, respectively. The 2D ^{13}C-^{1}H HSQC spectrum showed the ^{13}C chemical shifts, correlating with the ^{1}H chemical shifts expected for these residues. Most notably the 4-substituted C-4 value at δ 78.8, correlated with H-4 at δ 3.65, and C-3 at δ 80.4, correlating with H-3 at δ 3.85 ppm. The data observed in the 2D NMR spectra fit with the occurrence of these three types of residues.

In the δ 5.37 anomeric track the 2D ^{1}H-^{1}H COSY spectrum (**Supplementary Fig. S3**, green) showed H2 signals at δ 3.57 and 3.61, indicating the presence of at least two types of residue. The 2D ^{1}H-^{1}H TOCSY spectra revealed H-3 signals at δ 3.75 and 4.03, H-4 signals at δ 3.67 and 3.43, and an H-5 signal at δ 4.16, respectively. The signals for H-2 – H-4 at δ 3.57, 3.75, 3.43, respectively correspond with those expected for a terminal α-D-Glc*p*-(1→3)- unit [2, 3]. The H-5 signal for this unit is expected at δ ~4.02, which overlaps with the strong H-3 signal at that same chemical shift. The 2D ^{13}C-^{1}H HSQC spectrum (**Supplementary Fig. S3**) shows a C-5 (δ 72.4) and a C-3 value (δ 74.3) correlating with the ^{1}H chemical shift at δ 4.02, indicating the occurrence with H-5 at δ 4.02 as expected for the terminal α-D-Glc*p*-(1→3)- residue. The remaining combination of δ 3.61, 4.03, 3.67 and 4.16 for H-2 – H-5 are in line with a -(1→4)-α-D-Glc*p*-(1→3)- residue. The 2D ^{13}C-^{1}H HSQC spectrum (**Supplementary Fig. S3**) confirms the assignment of the ^{1}H chemical shifts, showing unsubstituted C-4 values δ 71.2, correlating with H-4 at δ 3.65, fitting with the 4-substituted α-D-Glc*p*-(1→3)- residue. Although no distinct signals were found for 3,4-disubstituted residues, there is a significant amount of terminal residues observed as indicated by the structural-reporter signals at δ 3.42 and 3.43 ppm, representing 8.4 % in relation to the anomeric signals. Since only (α1→4) and (α1→3) anomeric signals are

observed, the branched residues have to be 3,4-disubstituted. This is further supported by the methylation analysis data (**Table 2**) indicating approximately 8 % 3,4-disubstituted residues in **F1**.

Notably, the (α1→3)-anomeric track showed no H-3 signals at δ 3.85, whereas the 2D ^{13}C-^{1}H HSQC spectrum showed only 3-substituted C-3 (δ 80.4) correlating with H-3 at δ 3.85, and the 2D ^{1}H-^{1}H ROESY spectrum (**Supplementary Fig. S3**, red) showed inter-residual correlations between the (α1→3)-anomeric signal only with H-3 at δ 3.85, indicating that no sequential (α1→3)-linkages occur. Moreover, the 4-substituted C-4:H-4 signal is only observed at δ 78.8:3.65-3.67 ppm.

Smith degradation analysis

In order to confirm the absence of consecutive (α1→3)-linkages a sample of **F1** was subjected to Smith degradation with $NaIO_4$ under mildly acidic conditions, followed by reduction with $NaBH_4$ and mild hydrolysis with formic acid. Considering the linkage analysis, fragments of [α-D-Glc*p*-(1→3)-]$_n$α-D-Glc*p*-(1→2)-L-erythritol are to be expected, and due to over-hydrolysis erythritol and [α-D-Glc*p*-(1→3)-]$_n$D-Glc*p* fragments. HPAEC-PAD analysis (**Supplementary Fig. S4**) showed fragment peaks between 2 and 5 min elution time. Since fragments of [α-D-Glc*p*-(1→3)-]$_n$α-D-Glc*p*-(1→2)-L-erythritol and [α-D-Glc*p*-(1→3)-]$_n$D-Glc*p* with n≥1 are expected at retention times above 10 min [2] these results support the suggested absence of consecutive (α1→3)-linkages.

*Constructing a composite model for **F1***

The structural reporter signals at δ 3.43 and 3.42, for α-D-Glcp-(1→3)- and α-D-Glcp-(1→4)-residues, respectively in the 1D ^{1}H NMR spectrum of **F1** indicate that 8.4 % branching occurs. The relative intensities of the two distinctive peaks are equal, indicating 4.2 % α-D-Glcp-(1→3)- residues and 4.2 % α-D-Glcp-(1→4)- residues. Taking into account that there are no consecutive (α1→3)-linked residues, all 3-substituted residues must be (α1→4)-linked. Also the branched residues are (1→3,4)-α-D-Glcp-(1→4)- residues, amounting to 8.4 %. Since 40 % (α1→3) linkages are observed 31.6 % of the residues have to be –(1→3)-α-D-Glcp-(1→4)- residues. Since 4.2 % of (α1→3)-linked residues are terminal, there are 35.8 % -(1→4)-α-D-Glcp-(1→3)- residues. This leaves 15.8 % -(1→4)-α-D-Glcp-(1→4)- residues. The various data obtained from NMR spectroscopy, methylation analysis and Smith degradation analysis were combined to formulate the composite structure depicted in **Fig. 5** , showing all the identified structural elements in their correct relative abundance.

SUPPLEMENTARY TABLES

TABLE S1. List of *L. fermentum* NCC 2970 carbohydrate active enzymes and their related functional annotation as identified using the automated Carbohydrate-active enzyme Annotation (dbCAN).

CaZy family	Gene ID	Functional annotation	E-value
**AA1**	CDS0468	YbaK/prolyl-tRNA synthetase associated region	2.90E-18
**CBM50**	CDS0864	Mannosyl-glycoprotein endo-beta-N-acetylglucosamidase	6.10E-71
**CBM67**	CDS0598	3.2.1.40: Alpha-L-rhamnosidase	4.30E-32
**CE9**	CDS0218	3.5.2.3: Dihydroorotase<br>3.5.2.5: Allantoinase	3.50E-18
**GH13**	CDS1589	3.2.1.70: Alpha-glucosidase	1.40E-80
**GH2**	CDS0592	Beta-galactosidase	8.90E-90
**GH2**	CDS1127	3.2.1.23: Beta-galactosidase large subunit	4.30E-145
**GH2**	CDS1128	3.2.1.23: Beta-galactosidase	3.40E-25
**GH3**	CDS0597	3.2.1.52: Beta-N-acetylhexosaminidase	3.00E-47
**GH32**	CDS0603	Beta-fructofuranosidase	2.70E-73
**GH36**	CDS0833	Alpha-galactosidase	4.00E-278
**GH43**	CDS0594	3.2.1.72: Xylan 1,3-beta-xylosidase	4.00E-50
**GH65**	CDS0916	2.4.1.8: Glycosyl hydrolase family 65 protein	2.30E-149
**GH70**	CDS0221	2.4.1.5: dextransucrase	6.80E-289
**GH73**	CDS0359	hypothetical protein	6.30E-34
**GH73**	CDS0432	hypothetical protein	2.90E-36
**GH73**	CDS0495	N-acetylmuramidase	1.40E-38
**GH73**	CDS0630	Mannosyl-glycoprotein endo-beta-N-acetylglucosamidase	2.10E-35
**GH73**	CDS0864	Mannosyl-glycoprotein endo-beta-N-acetylglucosamidase	1.20E-36
**GH78**	CDS0598	3.2.1.40: Alpha-L-rhamnosidase	1.20E-166
**GT14**	CDS0985	hypothetical protein	2.70E-32
**GT2**	CDS0424	2.4.1.287: Rhamnopyranosyl-N-acetylglucosaminyl-diphospho-decaprenol beta-1,3/1,4-galactofuranosyltransferase	2.40E-24
**GT2**	CDS0427	2.4.2.53: Glycosyltransferase, group 2 family protein	6.10E-21
**GT2**	CDS0429	2.4.2.53: Glycosyl transferase family 2	5.30E-31
**GT28**	CDS1437	2.4.1.227 / 2.4.2.8: UDP-N-acetylglucosamine--N-acetylmuramyl-(pentapeptide) pyrophosphoryl-undecaprenol N-acetylglucosamine transferase	4.30E-52
**GT4**	CDS0404	2.4.1.208 / 2.4.1.290: Glycosyl transferase group 1	3.20E-20
**GT4**	CDS0405	2.4.1.208/ 2.4.1.241: Glycosyl transferase group 1	1.10E-31
**GT4**	CDS0942	2.4.1.- / 2.4.1.52: Glycosyltransferase Gtf1	8.90E-32
**GT4**	CDS0943	2.4.1.52: Glycosyltransferase	3.60E-26
**GT51**	CDS0197	2.4.1.129: Peptidoglycan glycosyltransferase	3.80E-61
**GT51**	CDS0346	2.4.1.129: Penicillin binding protein 2A	1.20E-63
**GT83**	CDS1403	hypothetical protein	8.10E-37

TABLE S2. Protein sequences identified via a BLASTp search using the *L. fermentum* NCC 2970 GH70 protein (CDS0221) as query. The sequence corresponding to the *L. reuteri* 121 GtfB 4,6-α-glucanotransferase is highlighted in bold.

NCBI protein names	Organism	Coverage	Identity	Length	Accession
Dextransucrase	*Lactobacillus fermentum* 39	100%	79%	1478	KLD54475.1
Dextransucrase	*Lactobacillus reuteri* mlc3	99%	66%	1488	WP_019251413.1
Dextransucrase	*Lactobacillus reuteri* JCM 1112	99%	66%	1488	WP_003668618.1
Dextransucrase	*Lactobacillus reuteri* DSM 20016	87%	66%	1363	ABQ83597.1
Dextransucrase	*Lactobacillus sanfranciscensis* DSM 20451	86%	69%	1151	KRM78746.1
Dextransucrase	*Lactobacillus fermentum* ATCC 14931	86%	79%	1014	WP_003683900.1
Dextransucrase	*Lactobacillus fermentum* 28-3-CHN	78%	78%	986	WP_004563243.1
Glycosyl hydrolase family 70	*Lactobacillus delbrueckii* subsp. *delbrueckii* DSM 20074 = JCM 1012	66%	73%	1294	WP_057717954.1
Inactive glucansucrase	*Lactobacillus salivarius* GJ-24	60%	88%	1626	EGM52218.1
Dextransucrase	*Lactobacillus delbrueckii* subsp. *lactis*	66%	73%	1294	WP_013439942.1
Glycosyl hydrolase family 70	*Lactobacillus salivarius* GJ-24	53%	88%	852	WP_050809355.1
Glycosyl hydrolase family 70	*Lactobacillus delbrueckii* subsp. *lactis* DSM 20072	62%	76%	1252	WP_057727099.1
Glycosyl hydrolase family 70	*Lactobacillus plantarum* AG30	57%	81%	932	WP_033607967.1
Glycosyl hydrolase family 70	*Lactobacillus delbrueckii* subsp. *lactis* DSM 20072	59%	78%	1210	WP_035182758.1
Glycosyl hydrolase family 70	*Lactobacillus delbrueckii* subsp. *lactis* DSM 20072	58%	80%	997	WP_025895575.1
Glycosyl hydrolase family 70	*Lactobacillus plantarum* subsp. *argentoratensis* DSM 16365	55%	84%	908	WP_057717369.1
Dextransucrase	*Lactobacillus plantarum* subsp. *argentoratensis* DSM 16365	54%	85%	880	KRL97820.1
Glycosyl hydrolase family 70	*Lactobacillus delbrueckii* subsp. *bulgaricus* ND02	56%	83%	954	WP_035171046.1
Glycosyl hydrolase 70 family protein	*Lactobacillus delbrueckii* subsp. *lactis* CRL581	54%	85%	922	EPB98082.1
Glycosyl hydrolase family 70	*Lactobacillus plantarum* WLPL04	56%	82%	922	WP_057138784.1
Glycosyl hydrolase 70 family protein	*Pediococcus pentosaceus* IE-3	57%	81%	926	WP_002833996.1
Glycosyl hydrolase family 70	*Lactobacillus delbrueckii* KACC 13439	56%	83%	965	WP_052933722.1
Glycosyl hydrolase family 70	*Lactobacillus delbrueckii* subsp. *delbrueckii* KACC 13439	54%	85%	922	KNZ37797.1
Cell wall-binding repeat protein	*Lactobacillus delbrueckii* subsp. *bulgaricus* PB2003/044-T3-4	58%	80%	957	EFK31460.1
Dextransucrase	*Pediococcus pentosaceus* DSM 20336	53%	85%	874	KRN47461.1
Glycosyl hydrolase family 70	*Lactobacillus delbrueckii* JCM 17838	54%	85%	922	WP_050952694.1
Glycosyl hydrolase family 70	*Lactobacillus delbrueckii* subsp. *lactis*	57%	85%	922	WP_035162295.1
Glycosyl hydrolase family 70	*Pediococcus pentosaceus* DSM 20336	54%	84%	883	WP_056979574.1
Dextransucrase	*Lactobacillus delbrueckii* subsp. *jakobsenii* ZN7a-9 = DSM 26046	54%	80%	966	WP_002879779.1
Glycosyl hydrolase family 70	*Lactobacillus delbrueckii* subsp. *jakobsenii* ZN7a-9 = DSM 26046	53%	80%	948	WP_057709472.1
Glycosyl hydrolase family 70	*Lactobacillus mucosae* LM1	53%	84%	881	WP_053069107.1
Dextransucrase	*Lactobacillus delbrueckii* subsp. *jakobsenii* ZN7a-9 = DSM 26046	53%	85%	895	KRO17768.1
Glycosyl hydrolase family 70	*Leuconostoc mesenteroides* 406	53%	85%	850	WP_059442690.1
**Inactive glucansucrase**	***Lactobacillus reuteri* 121**	**57%**	**77%**	**1619**	**AAU08014.2**
Putative glucansucrase	*Lactobacillus reuteri* ML1	59%	77%	1620	AAU08003.2
FIG00744899: Hypothetical protein	*Lactobacillus reuteri* pg-3b	62%	76%	1622	CUR36485.1
Hypothetical protein HQ33_10125	*Lactobacillus reuteri* TMW1.656	59%	77%	1602	KOF04763.1
Putative glucansucrase	*Lactobacillus reuteri* TMW1.106	53%	77%	1383	ABP88725.1
Putative dextransucrase	*Lactobacillus plantarum* 16	86%	52%	1348	WP_016526729.1
Hypothetical protein	*Lactobacillus sanfranciscensis* DSM 20451	57%	67%	924	WP_056958823.1
Inactive glucansucrase	*Lactobacillus panis* DSM 6035	57%	67%	1603	KRM25865.1
Hypothetical protein	*Pediococcus argentinicus* DSM 23026	53%	70%	898	WP_057799472.1
Dextransucrase	*Pediococcus argentinicus* DSM 23026	53%	70%	890	KRO24973.1
Dextransucrase	*Lactobacillus paraplantarum* DSM 10667	57%	65%	920	KRL44364.1
Hypothetical protein	*Lactobacillus plantarum* NL42	57%	65%	907	WP_052697219.1
Dextransucrase	*Lactobacillus paraplantarum* DSM 10667	57%	65%	929	WP_056988774.1
Hypothetical protein	*Lactobacillus plantarum* CIP104448	69%	47%	1266	WP_052661628.1
Hypothetical protein	*Lactobacillus acidipiscis* KCTC 13900	63%	49%	1567	WP_050955745.1
Hypothetical protein	*Lactobacillus acidipiscis* DSM 15353	60%	51%	1139	WP_056988183.1
Dextransucrase	*Lactobacillus acidipiscis* DSM 15353	60%	51%	1143	KRN79505.1
Dextransucrase	*Lactobacillus aviarius* subsp. *aviarius* DSM 20655	99%	50%	1567	KRM39240.1

TABLE S3. ^{1}H and ^{13}C chemical shifts determined from 1D and 2D NMR spectroscopy for *L. fermentum* NCC 2970 GtfB polymer product fraction **F1**. Residue labels **A**, **B**, **C**, **D**, **E** and **F** correspond with those used in Fig. 5.

	**A**		**B**		**C**		**D**		**E**		**F**	
	^{1}H	^{13}C	^{1}H	^{13}C	^{1}H	^{13}C	^{1}H	^{13}C	^{1}H	^{13}C	^{1}H	^{13}C
1	5.41	100.6	5.41	100.6	5.37	100.0	5.41	100.6	5.41	100.6	5.37	100.0
2	3.63	72.3	3.69	71.3	3.61	72.3	3.69	71.3	3.59	72.3	3.57	72.3
3	3.97	74.2	3.85	80.4	4.03	74.2	3.85	80.4	3.70	73.5	3.75	73.5
4	3.65	77.8	3.66	71.3	3.67	77.8	3.66	77.8	3.42	70.3	3.43	70.3
5	3.85	72.0	3.75	73.5	4.16	71.3	3.85	72.0	3.75	73.5	4.02	72.9
6a	3.87	61.3	3.87	61.3	3.87	61.3	3.87	61.3	3.87	61.3	3.87	61.3
6b	3.77		3.77		3.77		3.77		3.77		3.77	

SUPPLEMENTARY FIGURES

FIG. S1. SDS-PAGE analysis of *L. fermentum* NCC 2970 GtfB GH70 protein samples at different stages of purification. Lane M, molecular mass standards; lane 1, sample of *E. coli* cell free extract; lane 2, sample of the insoluble fraction after centrifugation of lysed cells; lane 3, pooled fractions after Ni-NTA agarose column chromatography; lane 4, purified GtfB 4,3-α-glucanotransferase after anion-exchange Hi-trap column chromatography. Bands corresponding to the *L. fermentum* NCC 2970 GtfB protein are marked with an arrow.

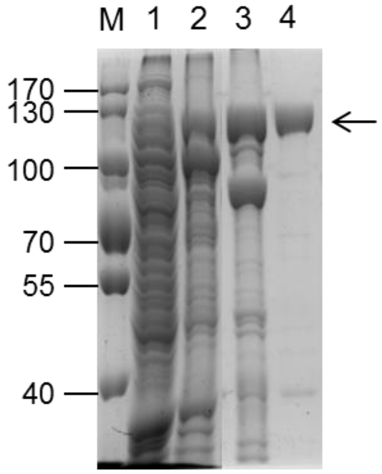

FIG. S2. Biochemical properties of the purified *L. fermentum* NCC 2970 GtfB enzyme. (**a**) Effect of pH on GtfB activity. Experiments were performed at 40°C and the relative enzyme activity was compared with that at pH 5.5 (100% value). (**b**) Effect of temperature on GtfB activity. The assays were carried out at pH 5.5 and relative enzyme activity was compared with that at 50°C (100% value). (**c**) Effect of temperature on GtfB stability. GtfB enzyme (0.1 mg ml^{-1}) was incubated for 10 min at the indicated temperature in 20 mM Tris–HCl pH 8.0 buffer containing 1 mM $CaCl_2$. Residual activity was assayed at 40°C using amylose V as substrate under the standard conditions described in the experimental section. Experiments were performed in triplicate, and the bars indicate the standard error of three replicates.

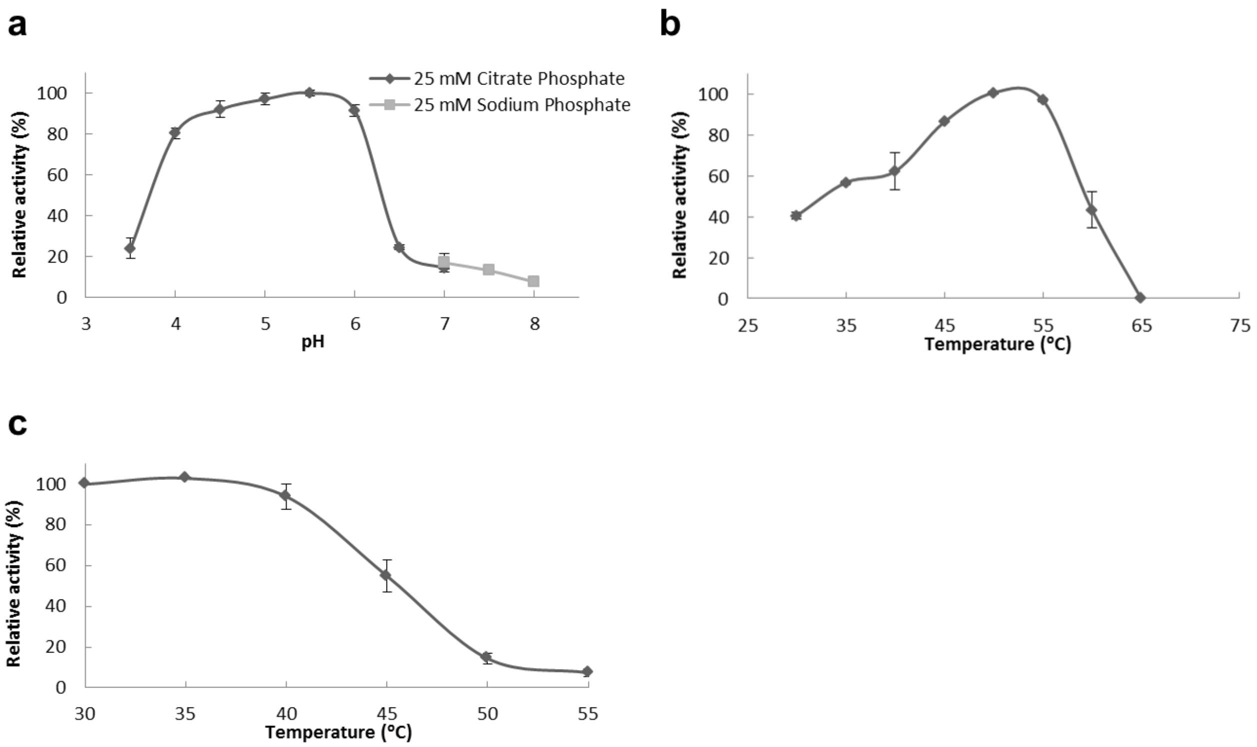

FIG. S3. 500-MHz 1D 1HNMR spectrum, 2D ^{1}H-^{1}H COSY (green), TOCSY spectra (mixing time 150 ms, grey), ROESY (red) and 2D ^{13}C-^{1}H HSQC spectrum of the Bio-Gel P2 polysaccharide fraction **F1**, obtained after 24 h incubation of 0.6% (w v^{-1}) amylose V with the *L. fermentum* NCC 2970 GtfB enzyme (25 µg ml^{-1}) recorded at 300K in D_2O. Peaks for (α1→4) and (α1→3) anomeric signals have been indicated. Structural-reporter signals are indicated.

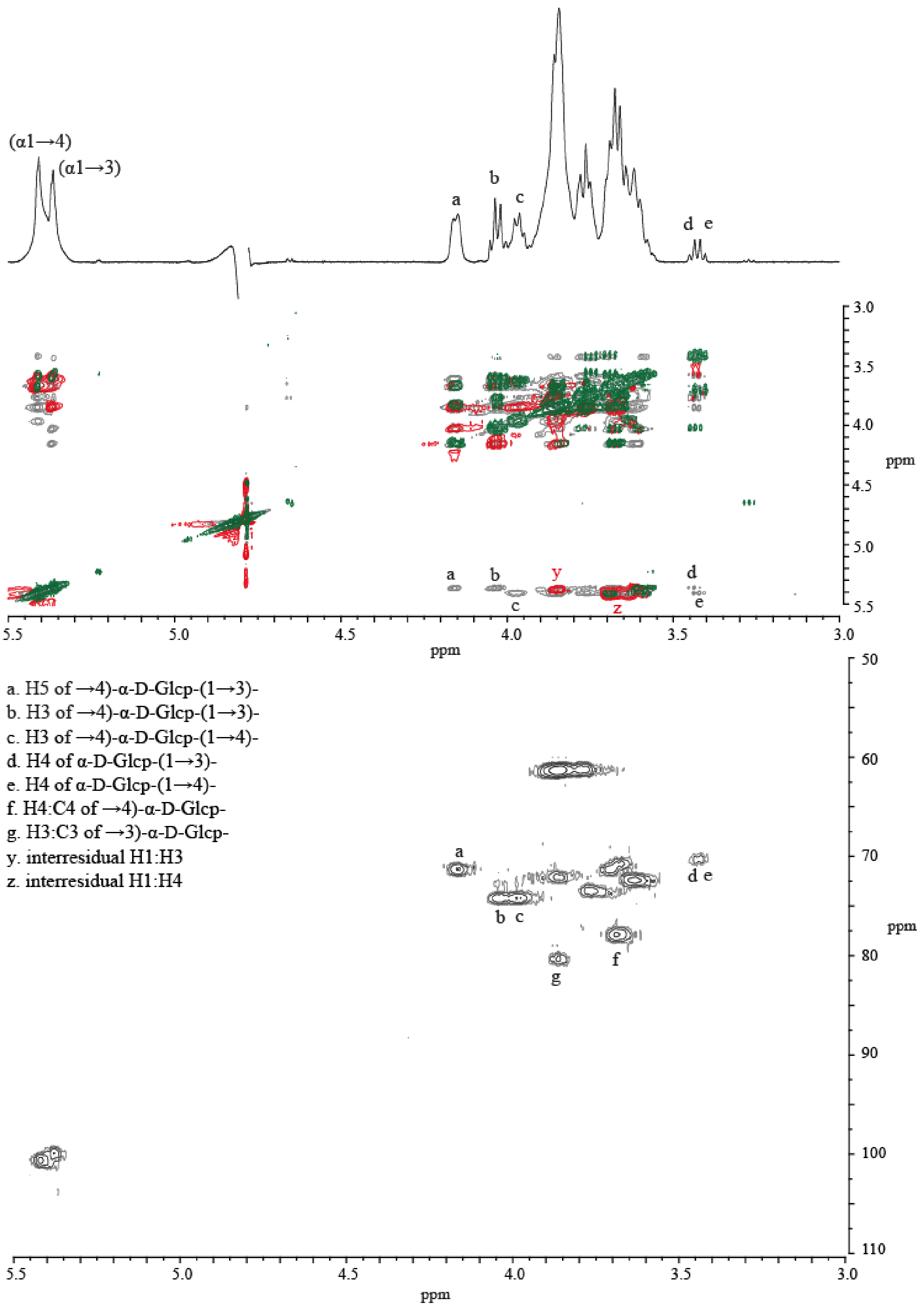

FIG. S4. HPAEC-PAD profile of the *L. fermentum* NCC 2970 GtfB product after Smith degradation. Eto, ethanediol, Gro, glycerol, Ero, erythritol, and Glc-Ero, glucosyl-erythritol.

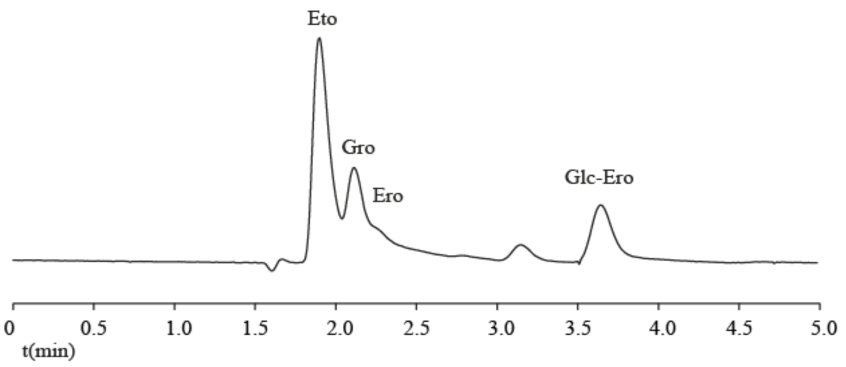

References

1. van Leeuwen, S. S., Kralj, S., Gerwig, G. J., Dijkhuizen, L. & Kamerling, J. P. Structural analysis of bioengineered alpha-D-glucan produced by a triple mutant of the Glucansucrase GTF180 enzyme from *Lactobacillus reuteri* strain 180: generation of (alpha1→4) linkages in a native (1→3)(1→6)-alpha-D-glucan. *Biomacromolecules* **9**, 2251-2258 (2008).

2. van Leeuwen, S. S. *et al*. Structural analysis of the alpha-D-glucan (EPS180) produced by the *Lactobacillus reuteri* strain 180 glucansucrase GTF180 enzyme. *Carbohydr. Res.* **343**, 1237-1250 (2008).

3. van Leeuwen, S. S. *et al*. Structural analysis of the alpha-D-glucan (EPS35-5) produced by the *Lactobacillus reuteri* strain 35-5 glucansucrase GTFA enzyme. *Carbohydr. Res.* **343**, 1251-1265 (2008).
